# Efficacy and Safety of Extracorporeal Shock Wave Therapy and 1064 nm Nd:YAG Laser in Managing Nodules and Edema Following Cosmetic Injections: A Case Series and Systematic Literature Review

**DOI:** 10.1111/jocd.70883

**Published:** 2026-05-05

**Authors:** Jieyi Wang, Jiaxu Gu, Zhuoying Wang, Peishi Yin, Xingling Jian, Bo Yu

**Affiliations:** ^1^ Department of Dermatology Peking University Shenzhen Hospital Shenzhen China; ^2^ Department of Dermatology Shenzhen Xinhua Hospital Shenzhen China; ^3^ St. Anne's College University of Oxford Oxford UK; ^4^ Peking University Health Science Center Peking University Beijing China; ^5^ Shenzhen Key Laboratory for Translational Medicine of Dermatology, Biomedical Research Institute Shenzhen Peking University—The Hong Kong University of Science and Technology Medical Center Shenzhen China

**Keywords:** cosmetic injection, energy‐based devices, nodules, swelling, therapy

## Abstract

**Background:**

Advances in injectable techniques and materials have driven rapid growth in the esthetic injection market, with more products showing excellent efficacy and safety. Nevertheless, adverse reactions continue to be reported, among which chronic inflammatory nodules and swelling are particularly challenging to manage. Despite many etiological theories and diagnostic consensus, clinical treatment remains complex and difficult. Novel approaches are continually being explored. Among them, energy‐based devices (EBDs) therapy has shown promising potential and clinical value, as evidenced by some clinical reports.

**Objective:**

Evaluate the efficacy and safety of extracorporeal shock wave therapy (ESWT) and 1064 nm Nd:YAG laser in managing adverse reactions such as nodules and swelling following cosmetic injections.

**Methods:**

This study demonstrates four treatment cases combining different energy‐based devices, documenting therapeutic protocols and efficacy. A systematic literature review is incorporated to evaluate EBDs applications and therapeutic value.

**Results:**

All of the four cases recovered from nodules or swelling without side effects by receiving ESWT and 1064 nm Nd:YAG laser, and the existing reports have demonstrated preliminary clinical experience with EBDs, showing promising efficacy and potential for broader applications in this field.

**Conclusion:**

ESWT and 1064 nm Nd:YAG laser have demonstrated effectiveness in treating post‐injection nodules and swelling, achieving high patient satisfaction with minimal side effects. Future studies should focus on elucidating their mechanisms and standardizing treatment protocols. Other energy‐based devices (EBDs) may have potential in this field, which requires further clinical verification.

## 1 Introduction

The global injectable esthetics market has expanded significantly, showing consistent growth in mesotherapy, filler injection, and botulinum toxin injection procedure numbers [1]. Advances in material science and molecular biology have enabled widespread application of synthetic polymers for esthetic injection medicine [2, 3], not only is Hyaluronic acid (HA) widely recognized for its effectiveness, various non‐HA soft tissue fillers such as polycaprolactone (PCL), polymethylmethacrylate (PMMA), poly‐l‐lactic acid (PLLA), calcium hydroxyapatite (CaHA), polyvinyl alcohol (PVA) and others have also become attractive options [4]. Although these materials are designed for biocompatibility, adverse events (AEs) may still occur due to individual variations, material impurities, injection techniques, or other factors [5]. Notably, chronic nodules and swelling present clinical challenges, compromising esthetic outcomes and creating unnecessary economic burdens. Such symptoms are typically diagnosed as foreign body granulomas (FBG) or delayed‐onset reactions (DOR). Several pathogenic mechanisms have been proposed, including autoimmune responses [6], material immunogenicity, biofilm formation [7], and the “adjuvant” effect [8]. Current treatments include intralesional corticosteroid injections, hyaluronidase, systemic corticosteroids, systemic immunosuppressants, empirical antibiotics, and surgical excision. However, limited efficacy and side effects persist. Consequently, researchers continue to explore more efficient and rapid approaches through clinical practice.

Studies have shown that energy transduction can activate multiple intracellular signaling pathways, modulating cellular processes such as proliferation, tissue repair, regeneration, and inflammation suppression [9]. Therefore, energy‐based devices (EBDs) have become indispensable tools in dermatology, offering minimally invasive or non‐invasive treatment options for a wide range of facial rejuvenation and body contouring needs [10]. These devices utilize various forms of energy, including laser, intense pulsed light (IPL), radiofrequency (RF), high‐Intensity focused ultrasound (HIFU), Extracorporeal shock wave therapy (ESWT) and cryolipolysis—to target specific tissues with precision while minimizing damage to surrounding structures. Beyond their esthetic applications, EBDs have proven clinically valuable in dermatosis therapeutics with well‐documented efficacy in scar management [11, 12], vascular lesions (telangiectasias/hemangiomas), and inflammatory conditions (acne vulgaris/eczematous dermatoses). The mechanisms include effectively reducing inflammatory mediators, inhibiting pathological neovascularization, and promoting cutaneous wound healing through enhanced collagen synthesis and extracellular matrix remodeling [13]. It is worth noting that application of EBDs has expanded to include management of AEs associated with cosmetic injections with promising clinical results. Previous studies have reviewed laser‐assisted therapies for managing AEs following injectable cosmetic procedures [14]. However, these researches have primarily focused on invasive laser treatments. With continuous advancements in EBDs and expanded clinical applications, an increasingly diverse of energy modalities are now being employed. This article presents case reports and a systematic evaluation of updated clinical applications.

## 2 Materials and Methods

This study retrospectively analyzed data from four female patients who developed nodules and edema following cosmetic injections in the facial regions. The patients were treated between June 2022 and March 2024. All patients received preoperative screening for autoimmune and infectious diseases, with positive cases excluded. No participants were undergoing NSAIDs or corticosteroids during EBDs treatment. The therapeutic protocol comprised 1064 nm long‐pulsed Nd:YAG laser (Cynergy Multi‐Wavelength Platform, Cynosure) and ESWT (THERAPEUTIC LC‐580 system). Informed consent was obtained from all participants for analysis and publication. Besides, a systematic literature review was conducted to evaluate the consistency of existing evidence regarding the efficacy of EBDs in treating non‐infectious chronic inflammatory conditions such as nodules and swelling.

## 3 Case Reports

### 3.1 Case 1

**FIGURE 1:**
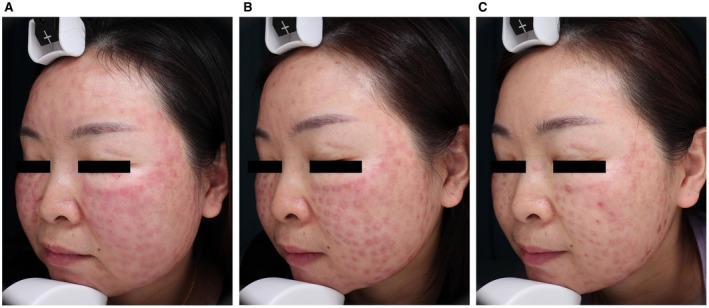
(A) Nodules and localized edema developed on the facial region. (B) Swelling markedly improved after 3 treatments. (C) Post final treatment, swelling resolved with residual post‐inflammatory erythema.

A 43‐year‐old woman (Fitzpatrick skin types IIII) developed nodules and swelling 1 week after receiving facial mesotherapy (mixed) at a cosmetic clinic. Despite 2 months of oral antibiotics, antihistamines, and hyaluronidase injections, her symptoms persisted. Upon presentation to our department, physical examination revealed firm facial nodules with generalized edema and histopathological examination confirmed FBG formation. She subsequently underwent ESWT using 15 and 20 mm handpieces for deep and superficial tissue targeting respectively, with parameters set at 1.6–2.0 bar energy and 4 Hz frequency; each full‐face session delivered 2000 pulses, administered twice weekly for 3 weeks. Final outcomes demonstrated marked improvement (Figure 1) with only localized erythema remaining. The patient reported satisfactory outcomes, and no recurrence occurred during 6 months of follow‐up.

### 3.2 Case 2

**FIGURE 2:**
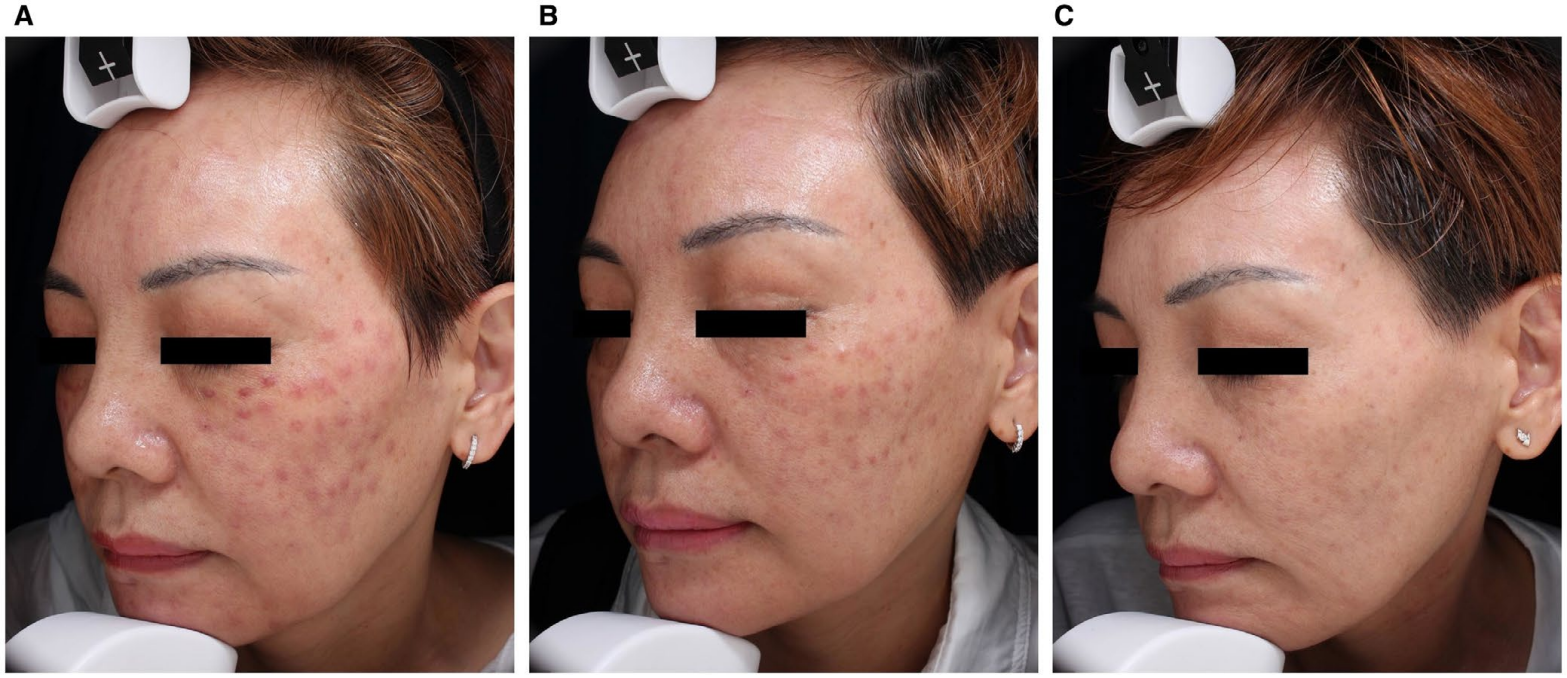
(A) Firm, well‐defined red nodules were observed on the bilateral cheeks. (B) Significant nodule regression was observed following three treatments. (C) Nodules have nearly disappeared completely after final treatment.

A 46‐year‐old woman (Fitzpatrick skin type VI) developed pruritic facial nodules following an unspecified “brightening” mesotherapy injection at a cosmetic clinic. Despite a 2‐week course of oral antihistamines and topical corticosteroids, her symptoms persisted. Clinical examination revealed erythematous nodules with localized telangiectasia, suggestive of foreign body granuloma (histopathology not obtained). Pre‐laser therapy, one ESWT session was administered to the lesion. Subsequent treatment employed a 1064 nm Nd:YAG laser (7 mm spot size, 35 ms pulse duration, and fluence of 55–65 J/cm^2^) every 2 weeks for 8 weeks, and remarkable resolution of nodules and erythema was observed after treatments (Figure 2), with excellent patient satisfaction and no recurrence or adverse effects observed during 3‐month follow‐up.

### 3.3 Case 3

**FIGURE 3:**
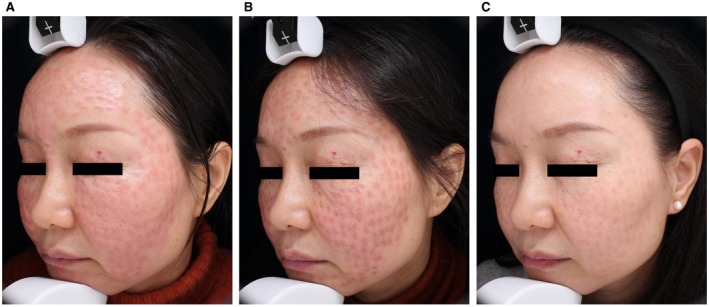
(A) Diffuse erythema and edema involving the entire face were observed. (B) Marked resolution of edema after one treatment, but significant erythema persisted. (C) The lesion completely resolved with only mild post‐inflammatory hyperpigmentation following three treatments.

A 47‐year‐old woman (Fitzpatrick skin type III) presented with persistent facial erythematous papules and edema following unspecified mesotherapy, unresponsive to 2 weeks of oral corticosteroids. Physical examination revealed confluent papules with swelling. She underwent three sessions of 1064‐nm Nd:YAG laser treatment (7 mm spot size, 30 ms pulse duration, 65–75 J/cm^2^) at 2‐week intervals. Remarkable improvement was observed with near‐complete resolution, leaving only mild post‐inflammatory erythema on the cheeks (Figure 3). The patient reported high satisfaction with no recurrence or adverse effects during the 2‐month follow‐up.

### 3.4 Case 4

**FIGURE 4:**
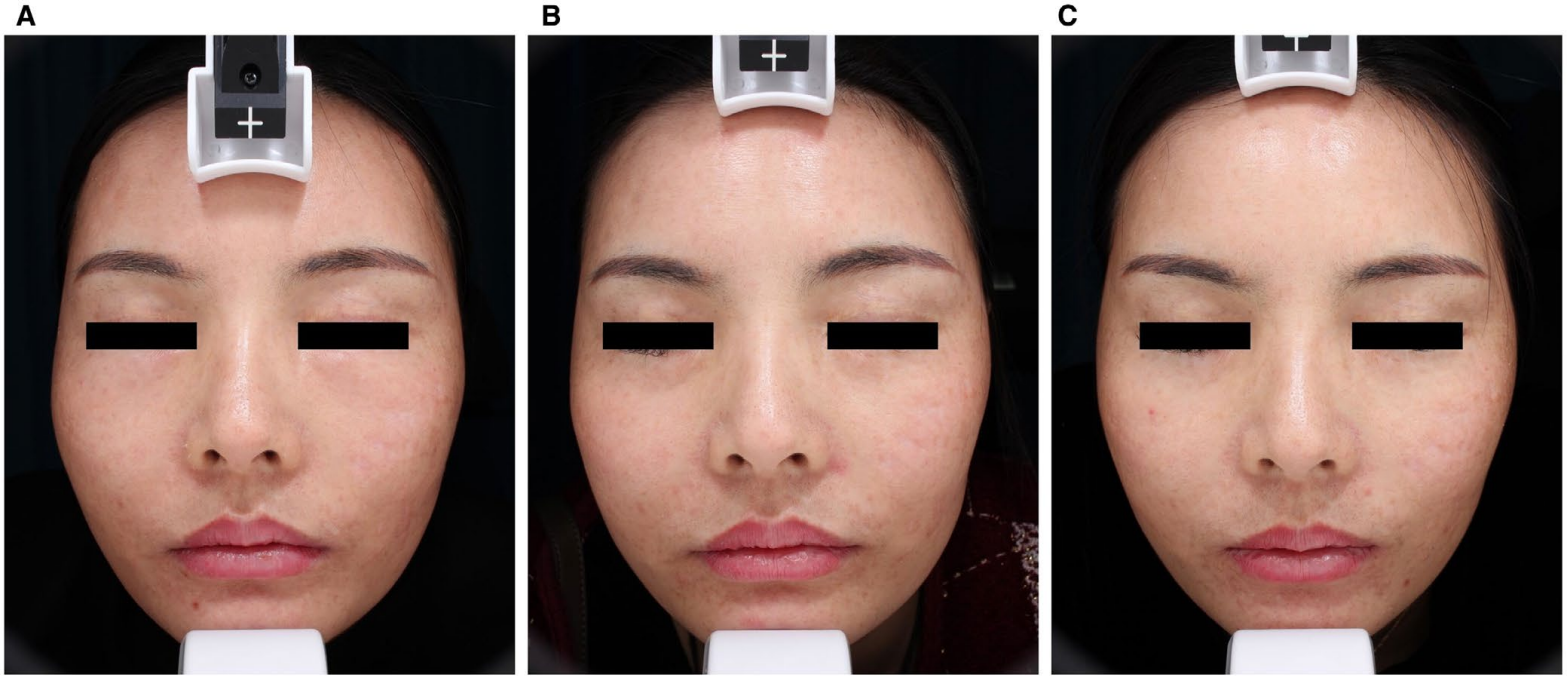
(A) Tissue edema was observed in the cheek and nasolabial fold area. (B) Improvement of edema after three treatments. (C) The lesion nearly resolved after eight treatments.

A 42‐year‐old woman received bilateral nasolabial fold augmentation with PLLA at a cosmetic clinic. Three months post‐procedure, she developed persistent nodules and swelling in the treated areas. Unresponsive to a 1‐month course of oral corticosteroids, the patient underwent histopathological examination, which confirmed FBG formation. Tofacitinib therapy (5 mg twice daily) initially achieved good clinical response but was discontinued after 1 month due to drug‐related dizziness, and symptom recurrence occurred 1 month after Tofacitinib cessation. ESWT was subsequently administered twice weekly for a total of 8 sessions using 15 and 20 mm handpieces at 1.5–1.8 bar pressure (1000 pulses per treatment area), achieving significant swelling reduction and decreased recurrence (Figure 4). The patient reported satisfactory outcomes without adverse effects and remains under treatment and follow‐up.

## 4 Results

In our reports, four patients diagnosed with FBG following cosmetic injections (confirmed by histopathology or clinical presentation) demonstrated poor response to conventional oral/topical therapies. After excluding infections, they received either 1064 nm laser therapy, ESWT, or a combination of both. All achieved favorable outcomes with high patient satisfaction, no reported recurrence, and no treatment‐related adverse effects. A literature review revealed that EBDs have demonstrated favorable therapeutic outcomes and high patient satisfaction in some cases, as summarized in our analysis (Table 1).

**TABLE 1:** Clinical applications of EBDs in managing cosmetic injection‐induced nodules and edema.

References	Patients	Filler material	Clinical feature	Diagnosis	Energy‐based device	Adjunct treatment	Sessions	Adverse effects	Efficacy
Cassuto et al. [15]	219 patients (204 women and 15 men, age 23–72 years)	Permanent fillers	Lumps or nodules	Inflammatory reactions	Invasive ILT 808 nm diode laser	Incision and drainage	1.7 sessions (average number)	Swelling, scar	Partial improvement (30%), complete resolution (62%), discontinuation (8%)
Hong et al. [16]	A 48‐year‐old woman	HA	Swelling, tenderness	FBG	Invasive intradermal bipolar RF	None	2 sessions (1‐week intervals)	None	Significant improvement
Schelke et al. [17]	242 patients (214 women and 28 men, range age 25–78 years)	Variety (PMMA predominates)	Lumps, nodules	Inflammatory reaction	Invasive ILT (810 and 1470 nm diode laser)	Manual compression	1–7 sessions	Inflammatory reactions	Improved (83%), resolved (9%), unknown (5%) and not improved (3%)
Hong et al. [18]	A 52‐year‐old woman	PLLA	Nodules, swelling	FBG	Noninvasive HIFU and QMR	None	8 sessions (1‐ week intervals)	None	Marked improvement
Ostezan et al. [19]	A 75‐year‐old woman and a 58‐year‐old woman	HA	Nodules	DOR	ESWT	None	4 and 3 sessions, respectively	None	Complete resolution
Hong et al. [20]	A 51‐year‐old woman	PCL	Nodules	FBG	Noninvasive HIFU and QMR	None	5 sessions (2‐week intervals)	None	Decreased size of mass
Zaccaria et al. [21]	181 patients (168 women and 13 men, mean age 49 years)	Permanent fillers	Nodules, swelling	Inflammatory reactions	Invasive ILT	Antibiotic therapy and incision	1.6 sessions (average number)	None	Complete resolution (38.7%), partial improvement (34.2%)
Piccolo et al. [22]	5 women (52–68 years)	HA	Nodules, edema	FBG	Invasive 1444 nm Nd:YAG laser	None	Once	None	Good to excellent improvement
Wang et al. [23]	7 women (29–34 years)	Mixed or unknown	Nodules	FBG	IPL	Intralesional corticosteroids	1–4 sessions (1‐month intervals)	None	Significant improvement
Goisis et al. [24]	9 women (age unknown)	PMMA	Erythema, nodules	FBG	ESWT	Surgery	6 sessions (2 sessions a week for 3 weeks)	None	Important improvement
Seo et al. [25]	A 42‐year‐old woman	PDLLA	Nodules	Noninflammatory nodules	Noninvasive monopolar RF	Strong compression	2 sessions	None	Complete resolution

Abbreviations: DOR, delayed‐onset reactions; ESWT, extracorporeal shock wave therapy; FBG, foreign body granuloma; GFRB, granulomatous foreign body response; HA, hyaluronic acid; HIFU, high‐intensity focused ultrasound; ILT, intralesional laser treatment; PCL, polycaprolactone; PDLLA, poly‐d,l‐lactic acid; PLLA, poly‐l‐lactic acid; PMMA, polymethylmethacrylate; QMR, quantum molecular resonance.

## 5 Discussion

Non‐infectious nodules and swelling represent one of the most persistent and therapeutically challenging adverse events following cosmetic injections, causing significant physical and psychological distress to patients. A study indicated that 87.1% of post‐injection skin lesions exhibited FBG histologically [26]. Post‐injection FBG is a kind of granulomatous tissue reaction, which is a response to the presence of non‐self tissues [27]. Although this process primarily manifests as a T‐cell‐mediated type IV hypersensitivity reaction [28], it also involves complex interactions with biofilm, macrophages, and other immune cells [29]. Early interventions like hyaluronidase, antibiotic therapy, or intralesional steroid can resolve some symptoms. However, chronic refractory nodules often resist conventional treatments. While surgical excision and aspiration techniques can remove some materials, their efficacy diminishes significantly over time due to the development of cyst or granulomatous tissues surrounding the fillers [14]. Laser therapies have emerged as an effective alternative, offering dual benefits of filler material removal and inflammation control. Studies indicate that bipolar or monopolar RF delivers high‐energy waves to the dermal or subcutaneous layers, where tissue resistance converts electrical energy into heat, generating localized thermal coagulation zones while sparing the epidermis and may mitigate fibrosis by modulating M2 phenotype macrophage polarization [30]. Similarly, HIFU can precisely target abnormal tissue, induce collagen denaturation and regeneration, and adjust treatment depth by selecting appropriate frequencies. When combined with QMR, this approach further reduces inflammatory cells and alleviates tissue edema [18, 20]. Additionally, ESWT mechanically disrupts non‐degradable fillers as well as exerting biological effects, such as suppressing the expression of TGF‐β1, α‐smooth muscle actin (α‐SMA), collagen‐I, and fibronectin, thereby exerting anti‐inflammatory and anti‐fibrotic effects, which have been demonstrated in studies on ESWT for scar treatment [31], and ESWT also softens firm tissue, facilitating subsequent surgical excision or aspiration. For invasive treatment, the diode laser and 1444 nm Nd:YAG laser target the granuloma interior in a minimally invasive manner, liquefying filler particles to promote absorption and accelerate healing.

Analysis of current evidence reveals that early EBDs for cosmetic injections AEs primarily used invasive thermal ablation techniques analogous to surgical excision [14]. Subsequent advancements used more non‐invasive focused energy modalities that utilize physical mechanisms and biological modulation to effectively accelerate filler degradation and absorption while mitigating inflammation and fibrosis. Our clinical experience suggests: (1) surgical or invasive laser excision remains optimal for large volume or permanent fillers removal, and (2) focused non‐invasive energy devices should be prioritized for multifocal or small volume injections (particularly mesotherapy) when conventional methods fail.

In summary, ESWT and 1064 nm Nd:YAG laser demonstrate robust therapeutic potential for managing post‐cosmetic injection granulomas and edema, with the advantages of minimal invasiveness, rapid recovery, and high patient acceptability. The core conclusions of this study are confined to these two modalities, as they are the only ones validated through our clinical cases. While other EBDs reported in the literature show preliminary promise, their lack of validation in our series underscores the need for future studies to directly compare different EBD modalities, optimize treatment parameters, and evaluate long‐term outcomes. Such efforts will help refine therapeutic protocols and provide more targeted clinical guidance for managing these challenging adverse events.

## Author Contributions

J.W. and J.G. collected and analyzed patient data and drafted the manuscript. B.Y. supervised the study design and data analysis. Z.W., P.Y., and X.J. reviewed and revised the manuscript. Z.W. additionally served as the materials science consultant. All authors critically reviewed the manuscript for intellectual content and approved the final version for publication.

## Funding

The study was approved by Shenzhen Sanming Project (No. SZSM202311029), Shenzhen Key Medical Discipline Construction Fund (No. SZXK040) and Study on the Mechanism of Novel Exosome‐functionalized Gel in Promoting the Repair of Diabetic Chronic Wounds by Regulating the Inflammatory Microenvironment (2024A1515220018, 2025A1515010947).

## Ethics Statement

The authors have nothing to report.

## Consent

All patients have provided written informed consent for the publication of their treatment information and pixelated clinical photographs associated with this study. All personal identifying information has been anonymized to protect patient privacy.

## Conflicts of Interest

The authors declare no conflicts of interest.

## Data Availability

B.Y. take responsibility for the integrity of the data. The original contributions presented in this study are included in the article. Further inquiries can be directed to the corresponding author.
